# Nonalcoholic Fatty Liver Disease—A Concise Review of Noninvasive Tests and Biomarkers

**DOI:** 10.3390/metabo12111073

**Published:** 2022-11-05

**Authors:** Tamara Bassal, Maamoun Basheer, Mariana Boulos, Nimer Assy

**Affiliations:** 1 Internal Medicine Department, Galilee Medical Center, Nahariya 2210001, Israel; 2 Azrieli Faculty of Medicine in the Galilee, Bar-Ilan University, Safed 1311502, Israel

**Keywords:** nonalcoholic fatty liver disease (NAFLD), metabolic-associated fatty liver disease (MAFLD), nonalcoholic steatohepatitis (NASH), advanced fibrosis, liver cancer, metabolic syndrome, non-invasive test (NIT), biomarkers

## Abstract

Non-alcoholic fatty liver disease (NAFLD) is the most common liver disease worldwide, with a continuously growing prevalence. The pathophysiology of the disease is complex and includes several mechanisms, with metabolic syndrome and insulin resistance playing a major role. It is crucial to diagnose NAFLD before it advances to nonalcoholic steatohepatitis (NASH), which can progress to cirrhosis, presented by its complications which include ascites, portal hypertension, bleeding varices and encephalopathy. Another important complication of NAFLD and cirrhosis is hepatocellular carcinoma (HCC), a cancer with increasing incidence and poor prognosis. Even with the growing prevalence of NAFLD, diagnosis via liver biopsies is unrealistic, considering the costs and complications. Noninvasive tests, including serum biomarkers and elastography, are cost-effective and convenient, thereby replacing liver biopsies in diagnosing and excluding liver fibrosis. However, currently, these noninvasive tests have several limitations, such as variability, inadequate accuracy and risk factors for error. The limitations and variability of these tests comet the investigator to propose combining them in diagnostic algorithms to produce more accurate tools. Identifying patients with significant fibrosis is important for targeted therapies to prevent disease progression. Effective screening using noninvasive tests can be crucial for patient risk stratification and early diagnosis.

## 1. Introduction

Non-alcoholic fatty liver disease (NAFLD) is the most common chronic liver disorder, with an estimated global prevalence of 25% [1]. NAFLD has a bidirectional correlation with components of the metabolic syndrome. It often occurs together with type 2 diabetes, obesity, dyslipidemia, and hypertension, which constitute cardio-metabolic disease [1,2,3,4]. This led a panel of experts in 2020 to suggest that the nomenclature of NAFLD should be updated to metabolic-associated fatty liver disease (MAFLD) [3]. Patients with NAFLD can progress to the more severe form of non-alcoholic steatohepatitis (NASH). At this stage, the hepatocyte damage may lead to fibrosis and progress to cirrhosis in 20% of patients with NASH [5,6,7]. The exact mechanisms leading to NAFLD are not fully understood, but insulin resistance seems to play an important role [4].

NAFLD is defined by the presence of >5% of hepatic steatosis demonstrated, either radiographically or histologically, in the absence of significant alcohol consumption. The definitive diagnosis of NASH requires a liver biopsy with the histologic demonstration of >5% of hepatic steatosis in addition to evidence of hepatocyte inflammation in the form of ballooning degeneration [6,7]. Liver biopsy is considered the gold standard for the diagnosis of the global status of fibrosis of nonalcoholic fatty liver disease. Nevertheless, biopsies are invasive and carry the risk of complications and sampling errors, making biopsies impractical in being applied on a population level [8]. This has led to continuous searching for noninvasive biomarkers for diagnosing and staging liver fibrosis, especially in high-risk patients who benefit the most from early interventions to prevent progression to cirrhosis [9]. Among the imaging biomarkers used are Fibroscan and magnetic resonance imaging (MRI) [10]. Serum biomarkers include cytokeratin-18 (CK-18), a biomarker of apoptosis in NASH, liver enzymes and Pro-C3, a biomarker of fibrosis [11]. 

The aim of the study is to review the noninvasive tests used in the diagnosis and follow-up of patients with NAFLD. 

## 2. NAFLD Represents a Wide Spectrum of Diseases

In recent decades, NAFLD prevalence has increased into pandemic dimensions, affecting about one-third of western populations, probably due to the increasing prevalence of obesity and metabolic disease [12,13]. NAFLD represents a spectrum of diseases ranging from simple steatosis to NASH and, finally, cirrhosis. Although approximately only 10–20% of patients with NAFLD progress to NASH, and 15–20% of patients with NASH progress to cirrhosis, the absolute numbers are not negligible due to the wide prevalence of NAFLD [14]. The complications of NAFLD can be divided into hepatic and extra-hepatic. The hepatic complications of NAFLD include progression to cirrhosis. Studies have shown that the progression from steatosis to fibrosis can take 14 years, while the progression from NASH to cirrhosis may take 7 years [15]. Decompensated cirrhosis which can be manifested as hepatic encephalopathy, ascites or gastrointestinal bleeding secondary to esophageal varices, occurs annually at a rate of 3–4% in patients with compensated cirrhosis [16]. A meta-analysis of 13 studies showed higher mortality rates in patients with cirrhosis than in patients without fibrosis [17]. Hepatocellular carcinoma (HCC) is another serious complication of NAFLD, with poor outcomes and limited therapeutic options. The majority of NAFLD-related HCC cases occur in the setting of cirrhosis. However, about 20% of the cases occur in non-cirrhotic livers. An important risk factor for developing HCC is the presence of diabetes and metabolic syndrome [18,19]. A recent study revealed that the lack of fibroblast growth factor 21 (FGF21), which has anti-inflammatory effects, enhances the progression from NASH to HCC [20]. In addition to HCC, there is a higher incidence of extra-hepatic malignancies, particularly colorectal and uterine cancers, in which the risk is increased twofold [21]. It is not surprising that cardiovascular risk is increased in the presence of NAFLD since both of them share many risk factors, including dyslipidemia, obesity, hypertension and diabetes. A key factor in the development of both diseases is insulin resistance which contributes to the lack of lipolysis suppression, thereby increasing free fatty acids (FFA) in the circulation, ending up accumulating in the liver and blood vessels [22,23,24]. Cardiovascular complications are the most common cause of death in NAFLD, accounting for nearly 40% of the cases [25]. NAFLD is a risk factor for severe disease and liver injury [26,27].

## 3. Serum Biomarkers of NASH

Since NASH is currently the target of pharmacological treatment, it is important to develop new serum biomarkers for NAFLD and NASH diagnosis, to aid in identifying high-risk patients who require further follow-up and treatment.

### 3.1. Alanine Aminotransferase (ALT)

ALT levels are usually elevated in patients with NAFLD. The mechanism of ALT elevation is thought to be associated with lipotoxicity and increased hepatic triglyceride content [6]. However, no correlation was found between the degree of ALT elevation and the severity of fibrosis or liver inflammation [6,28]. Liver biopsies of patients with elevated ALT revealed similar rates of advanced fibrosis as for patients with normal ALT levels. Therefore, ALT levels are poor biomarkers in assessing the degree of disease progression and should not be used to guide clinicians in deciding to perform liver biopsies [29].

### 3.2. PRO-C3

Liver fibrosis is characterized by the accumulation of collagen proteins. Fragments of collagen, called propeptides, are released during the process of fibrosis and may be useful as biomarkers of fibrotic tissue formation in the liver [14]. The N-terminal propeptide of type 3 procollagen (PRO-C3) is released by protease A disintegrin and metalloproteinase with thrombospondin motifs 2 (ADAMTS-2) [30,31]. Serum concentrations of N-protease cleaved PIIINP neo-epitope (PRO-C3) are associated with the degree of liver fibrosis and can be used to monitor the response to treatment with antifibrotic agents [32,33]. A recent study measured PRO-C3 levels in patients with severe obesity before and after bariatric surgery. Furthermore, they compared liver histology from biopsies obtained during the surgery. The study found that PRO-C3 levels were associated with advanced liver fibrosis in patients with severe obesity. The levels of PRO-C3 decreased after the bariatric surgery, and the decrease was correlated with improvement in metabolic and liver parameters [34].

A PRO-C3-based score, ADAPT (**a**ge, presence of **d**i**a**betes, **P**ro-c3 and pla**t**elet count), which includes clinical and metabolic parameters, can predict the presence or absence of fibrosis in NAFLD and was shown to have high specificity and NPV. Recent studies have shown that the combination of ADAPT with liver stiffness measurements (LSM) can be reliably used to exclude advanced fibrosis in low-risk populations [33,35,36].

### 3.3. Cytokeratin-18 (CK-18)

CK-18 is an intermediate filament protein found in liver hepatocytes. It is cleaved and released from hepatocytes during apoptosis [37,38,39]. Therefore its serum concentration correlates with the degree of hepatocyte damage occurring during histological changes of NASH and NAFLD [20,21,22]. CK-18 concentrations are a marker of disease severity in NASH [40]. CK-18 was detected by ELISA, but a recent study suggests the use of new monoclonal antibodies raised against CK-18, which are more specific than the ELISA, in which the cut-off values were imprecise [41].

## 4. Noninvasive Fibrosis Scores in NAFLD

Noninvasive tests (NITs) can be used to exclude advanced fibrosis and to aid in risk stratification and specialist referral. NITs are considered ideal when they provide similar information to a biopsy, but none of these tests currently reach the accuracy of a liver biopsy. NITs of liver fibrosis have been suggested to be less accurate in type 2 diabetes mellitus (T2DM). A recent study by Boursier et al. compared the accuracy of NIT between 1051 NAFLD patients with and without T2DM. The study concluded that there is decreased accuracy of NIT in T2DM, which is partially attributed to the fact that T2DM modifies the level of some NAFLD biomarkers [42]. On the other hand, liver fibrosis biomarkers were found to be correlated positively with cardiovascular risk (CVR) scores. High CVR scores tend to be higher among patients with advanced fibrosis, supporting the association between liver fibrosis and cardiovascular risk [43].

### 4.1. Serum Scores

#### 4.1.1. Fibrosis-4 (FIB-4)

FIB-4 is a score used to assess liver fibrosis based on easily available and inexpensive parameters. The formula includes age, platelet count, aspartate transaminase (AST) and alanine transaminase (ALT) levels. FIB-4 was initially developed to assess liver fibrosis in patients who were co-infected with the human immunodeficiency virus (HIV) and hepatitis C virus (HCV) [44]. Shah et al. tested and validated its use in patients with NAFLD, in addition to comparing its performance to other markers of fibrosis [8,45]. A cut-off value of ≤1.3 can exclude advanced fibrosis in 90% of patients with NAFLD in primary clinics (high NPV). Values higher than 1.3 require additional workup in referral centers [46]. A study by Yun Hwa Roh et al. found that cut-off values of 2.68 showed acceptable PPV in predicting advanced fibrosis; the incorporation of this value with sonographic results increased the diagnostic accuracy of ruling in patients with advanced fibrosis [44]. In accordance with this finding, FIB-4 was also used for the diagnosis of fibrosis and not only for ruling it out. Despite the wide use of this score in excluding fibrosis, several limitations should be considered, such as its reduced specificity in older aged patients and the need for an adjusted threshold in patients aged ≥65, and that this score was developed and tested in populations with a higher prevalence of advanced fibrosis and not as a first-line screening tool [7,47].

Tsung-Po Chen et al. emphasize that fatty liver is prevalent in the ambulatory elderly. Age is a risk factor for advanced fibrosis, with the disease likely progressing from a steatotic to a fibrotic picture with age. However, there is no significant association between high-risk FIB-4 and BMI [48]. 

#### 4.1.2. Aspartate Aminotransferase/Platelet Ratio Index (APRI)

APRI is calculated as [(AST/ULN)/platelet count (109/L)] × 100. A cohort study of 145 patients with NAFLD showed that at the cutoff value of 1.0, APRI had a low sensitivity of 27% but a specificity of 89% [49]. This score has an excellent NPV for ruling out advanced fibrosis. Intermediate and high scores require further investigation, including liver biopsy [50]. A recent study by Yin-Lian Wu et al. (2021) evaluated the diagnostic performance of noninvasive scores, including APRI, in MAFLD. The definition of MAFLD was different from NAFLD and required no exclusion of other chronic liver diseases. Instead, MAFLD was defined as hepatic steatosis in the presence of metabolic disease. The NPV of APRI did not exceed 80%, and the PPV was as low as 50% at any cutoff value tested. These results led to the conclusion that APRI should not be used to evaluate advanced fibrosis in MAFLD [51].

#### 4.1.3. The NAFLD Fibrosis Score (NFS)

NFS consists of diabetes/impaired fasting glucose, age, AST, ALT, platelets, BMI and albumin [52]. According to a recent meta-analysis that compared the performance of several scores in diagnosing advanced fibrosis, the AUROC for NFS was 0.84. NFS has a high NPV (>90%) for excluding advanced fibrosis [53,54].

#### 4.1.4. Enhanced Liver Fibrosis (ELF)

ELF is a blood test that measures three markers of liver fibrosis: hyaluronic acid (HA), procollagen III amino-terminal peptide (PIIINP), and tissue inhibitor of matrix metalloproteinase 1 (TIMP-1) [55]. The cutoff value for diagnosing advanced fibrosis is ≥9.8, which demonstrated high specificity of >90% in diagnosing advanced fibrosis [56,57].

#### 4.1.5. The Fatty Liver Index (FLI)

FLI was suggested in 2006 by Bedogni et al. to aid physicians in selecting patients with suspected NAFLD to proceed to liver ultrasonography to confirm steatosis [58]. FLI is based on body mass index (BMI), waist circumference, triglycerides and gamma-glutamyltransferase (GGT). FLI can be calculated using many available online calculators. The results range from 0 to 100. FLI < 30 was ruled out, and FLI ≥ 60 ruled in hepatic steatosis [59].

##### Elastography

Elastography is a noninvasive imaging modality used to evaluate the degree of liver fibrosis [60]. Liver fibrosis or stiffness is the excessive accumulation of extracellular matrix proteins, including collagen, that occurs in most types of chronic liver diseases. The principle of elastography involves the transmission of acoustic waves through the liver parenchyma. The velocity of wave propagation within the parenchyma reflects the degree of stiffness or, in other words, the degree of fibrosis.

##### Transient Elastography (TE) (Fibroscan)

Using a special probe, Fibroscan can provide two parameters, the degree of steatosis measured by the controlled attenuation parameter (CAP) and the degree of fibrosis reflected by the liver stiffness measurement [61]. The liver volume measured by Fibroscan is 100 times bigger than a biopsy sample, thereby providing more precise information about the liver parenchyma [62].

Transient elastography (TE) < 10 kPa can rule out compensated advanced chronic liver disease (cACLD). Values between 10 and 15 kPa are suggestive of cACLD. Values > 15 kPa are highly suggestive of cACLD. Patients with chronic liver disease and an LSM < 10 kPa by TE have a negligible 3-year risk (<1%) of decompensation and liver-related death. A rule of 5 for LSM by TE (10–15–20–25 kPa) should be used to denote progressively higher relative risks of decompensation and liver-related death independently of the etiology of chronic liver disease (Table 1) [63].

##### Ultrasonographic Fatty Liver Index (US-FLI)

US-FLI was proposed by Ballestri et al. in 2012. The diagnosis of fatty liver was based on several ultrasonographic parameters, including the presence of liver-kidney contrast, graded as mild/moderate (score 2) and severe (score 3). Additional criteria include the presence (score 1 each) or absence (score 0 each) of posterior attenuation of an ultrasound beam, vessel blurring, difficult visualization of the gallbladder wall, difficult visualization of the diaphragm and areas of focal sparing. The US-FLI score ranges from 2 to 8. US-FLI ≥ 2 showed a good diagnostic performance to detect the minimum steatosis amount of 10% on histology with high sensitivity (90.1%) and specificity (90%) [64]. A study performed later by Xavier et al. demonstrated the significant superiority of US-FLI in discriminating between different grades of steatosis compared to the FLI score [65].

## 5. Metabolic Profile of NAFLD

Much research on NAFLD has been done, but no accurate mechanism has been described yet. A good understanding of the metabolic and biological mechanisms of this situation could help us in developing good predictors and markers. 

Several theories were suggested in the context of NASH development. The “double-hit” hypothesis suggests that the first insult or the “first hit” is characterized by lipid accumulation in the liver, resulting in steatosis, which enhances the liver’s susceptibility to the “second hit” characterized by inflammation and necrosis induced by oxidative stress and proinflammatory mediators [66,67]. The simplistic “double-hit” theory was substituted by a more complex theory, the “multiple-hit” hypothesis, which has become more accepted recently, representing the interaction of multiple metabolic and genetic factors leading to the development of NASH. Such “hits” include insulin resistance, hormones secreted from the adipose tissue, nutritional factors, gut microbiota and genetic and epigenetic factors [68].

### 5.1. The Role of Adipose Tissue in NAFLD

Obesity is associated with increased prevalence and severity of NAFLD. The adipose tissue functions as an endocrine organ secreting adipokines and cytokines [69]. Adiponectin is anti-inflammatory, and its levels are inversely associated with cardiovascular risk factors and visceral adiposity. It increases free fatty acid (FFA) oxidation and decreases gluconeogenesis resulting in decreased liver steatosis. Furthermore, it acts as a pro-inflammatory inhibitor, opposing the effects of tumor necrosis factor (TNF)-α and interleukin (IL)-6 [66,70,71]. Leptin secretion is directly proportional to the white fat mass. Circulating leptin levels are higher in patients with NAFLD than in healthy patients, and higher levels are associated with the severity [72]. Leptin has different effects depending on the stage of the disease. At early stages, it acts as an anti-steatotic and increases insulin sensitivity. At more advanced NAFLD stages, it has an unfavorable effect by promoting inflammation [73,74]. Pro-inflammatory cytokines are immunoregulatory cytokines that favor and enhance the process of inflammation [75]. Fat accumulation in the liver mediates cytokine production in hepatic cells. These cytokines induce inflammation, necrosis, cell apoptosis and mediated liver stiffness and fibrosis. Pro-inflammatory cytokines are also secreted by adipose tissue and are involved in the recruitment and activation of macrophages, a process that leads to chronic low-grade inflammation and the development of insulin resistance and cardiovascular diseases [73,74,75,76,77].

### 5.2. Lipotoxicity in NAFLD

Lipotoxicity is a leading factor in the development and progression of NAFLD and NASH. The total amount of triglycerides stored in the liver cells is not the main determinant of lipotoxicity [76,77]. Specific lipid classes, especially free fatty acids (FFA), which accumulate in hepatocyte cytoplasm, act as damaging agents via multiple mechanisms. These include activation of death receptors, modification of mitochondrial function and oxidative stress, which lead to the progression from NAFLD to NASH, its inflammatory form. Insulin resistance plays a major role in lipotoxicity by enhancing lipolysis in adipose tissue and increasing the amount of circulation FFAs [76,77].

### 5.3. The Role of Glucose in NAFLD

High-carbohydrate diets and NAFLD are strongly correlated [78]. Hyperglycemia is strongly associated with oxidative stress [79], leading to chronic inflammation and insulin resistance [80]. Insulin resistance in NAFLD is characterized by reductions in whole-body, hepatic, and adipose tissue insulin sensitivity. The mechanisms underlying the accumulation of fat in the liver may include excess dietary fat, increased delivery of free fatty acids to the liver, inadequate fatty acid oxidation, and increased de novo lipogenesis. Insulin resistance may enhance hepatic fat accumulation by increasing free fatty acid delivery and by hyperinsulinemia to stimulate anabolic processes. The impact of weight loss, metformin, and thiazolidinedione, all treatments aimed at improving insulin sensitivity, as well as other agents such as vitamin E, have been evaluated in patients with NAFLD and have shown some benefit. However, most intervention studies have been small and uncontrolled [81].

## 6. Risk Stratification

In light of the increased morbidity and mortality in NAFLD, and considering its wide prevalence, many efforts are put into identifying patients at risk for disease progression to enable early intervention and to prevent the development of cirrhosis [82]. 

**Figure 1:**
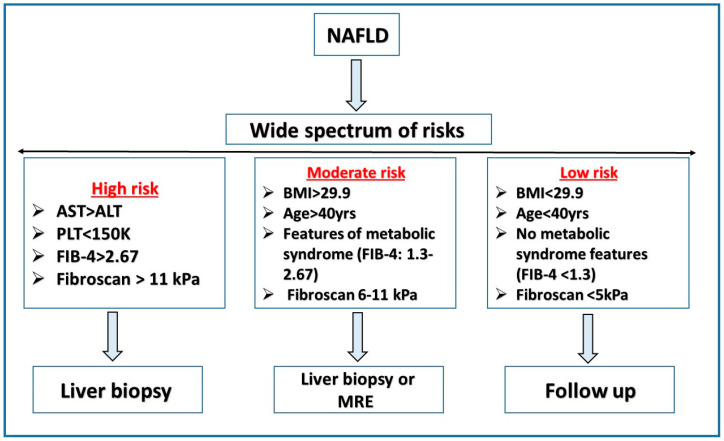
Management with NAFLD risks. Based on Raniella et al., 2016 [83].

Several algorithms were developed to screen NAFLD patients, each relying on different parameters (Figure 1) [83]. The European Association for Liver/-Diabetes/-Obesity Guidelines (EASL-EASD-EASO) uses the NFS and FIB-4 scores, while the German National Guidelines use LSM to guide the management of NAFLD patients [82].

NAFLD has almost reached epidemic proportions worldwide. Wide dissemination of current concepts on NAFLD and extensive collaboration between physicians, governments, non-governmental organizations, and the pharmaceutical industry is urgently needed to advance a NAFLD public health policies agenda that allows us to address this disease more holistically in a society-wide manner [80].

## 7. Future Perspective (Where the Field Should Go to Identify NAFLD)

Metabolomics and lipidomics studies help clinicians identify biomarkers associated with the pathophysiology of NAFLD and NASH. Impairment in amino acid metabolism in NAFLD is linked to insulin resistance and results in higher fasting concentrations of essential amino acids, as has also been observed in obesity. In patients with chronic liver disease, phenylalanine and its metabolite tyrosine are frequently found to be decreased, whereas BCAAs, especially leucine, isoleucine, valine and glutamate-glutamine are increased, mainly because of insulin resistance. However, as liver disease progresses, the opposite is often observed, with high amino acids (AAAs) and reduced BCAAs, especially in patients with chronic hepatic insufficiency. In addition, a reduction in hepatic glutathione levels and elevation of methionine levels has been associated with liver damage and the progression of NAFLD to NASH [82]. Another important observation is related to alterations in lipid metabolism. NASH is strongly associated with alterations in circulating fatty acid flux and intact lipids such as triglycerides and phospholipids, which is partially due to alterations in liver de novo lipogenesis (DNL), lipolysis rate and VLDL metabolism. Elevation in peripheral fatty acid flux and triglycerides containing fatty acids with low carbon number and double bonds, as well as a reduction in the levels of triglycerides containing polyunsaturated fatty acids (PUFAs), including ω-3 and ω-6 fatty acids, have been observed in NAFLD. Interestingly, this pattern of changes in triglycerides has also been reported in metabolic disorders, especially those associated with insulin resistance. Moreover, several studies have shown an increase in total bile acids in both liver and plasma of patients with NASH. In particular, a significant increase in fasting plasma concentration of bile acids, including glycocholic, taurocholic and taurochenodeoxycholic acid, in patients with NASH has been observed [84]. In order to derive robust metabolic signatures of NAFLD–NASH, the reported biomarkers will need to be consolidated and validated. Current efforts to improve metabolomics and lipidomics workflows are expected to improve our ability to effectively bridge the biomarker discovery stage, validation studies and translation to the clinic. Another area that is likely to lead to substantial advances in NASH biomarkers is the investigation of multiomic biomarkers. The gut microbiome is also a rich source of circulating metabolite biomarkers. Application of genome-scale metabolic modeling on shotgun metagenomics data from stool samples can predict which metabolites are being produced in specific diseases or physiological conditions. Such predictions can be validated by stool and serum metabolomics. Adding different layers of omics data is thus likely to lead to both improved sensitivity as well as specificity of NAFLD–NASH biomarkers [85].

Insulin resistance is the earliest detectable defect in the metabolic continuum leading to type 2 diabetes. Impaired glucose homeostasis is associated with obesity. This situation increased plasma-free fatty acids and ectopic lipids, which are connected to peripheral and hepatic insulin resistance. Branched-chain amino acids (BCAAs) are important nutrient signals that affect metabolism. Increased plasma levels of BCAAs are associated with a high risk of developing metabolic syndrome and insulin resistance. The significant down-regulation of genes related to BCAA catabolism and mitochondrial energy metabolism increased the expression of inflammation-related genes [84]. The branched-chain amino acids and microarray assay-detecting transcripts of these genes could be future tools for identifying NAFLD. 

The FibroScan-AST (FAST) score provides an efficient way to non-invasively identify patients at risk of progressive NASH for clinical trials or treatments when they become available, thereby reducing unnecessary liver biopsy in patients unlikely to have significant disease. FAST score performance is good across the full range of validation cohorts. AUROC ranged from 0.74 to 0.95, with PPV up to 0.85 and NPV ranging from 0.73 to 1 using the dual cutoffs approach, with cutoffs derived in the derivation cohort [86]. 

## 8. Conclusions

NAFLD is a multi-system disease with increasing prevalence. Early diagnosis contributes greatly to risk stratification and management, preventing the disease’s complications. Much research was done to develop and improve noninvasive tests which can replace liver biopsies and guide the management of NAFLD patients. Combining several tests and scores and creating charts for risk stratification and management helps the primary physician in managing such patients and in referring them to specialized centers. 

However, more research needs to be done to compare the best test combinations to increase the sensitivity of the diagnosis.

## Figures and Tables

**Table 1:** Fibroscan parameters used to determine the degree of steatosis and fibrosis reflected by the liver stiffness measurement. Abbreviations: LSM: liver stiffness measure, cACLD: compensated advanced chronic liver disease, CSPH: clinically significant portal hypertension.

LSM (kPa)	Liver Decompensation and Liver Related-Death
≤5	Exclude cACLD
10–15	+ Platelets ≥ 150 k → exclude CSPH ( avoid endoscopy)
15–20	+ Platelets ≥ 150 k → assume cACLD ( avoid endoscopy)
20–25	Highly assume cACLD
≥25	CSPH
